# Association of Reticular Pseudodrusen and Early Onset Drusen

**DOI:** 10.1155/2013/273085

**Published:** 2013-05-16

**Authors:** Flore De Bats, Benjamin Wolff, Martine Mauget-Faÿsse, Isabelle Meunier, Philippe Denis, Laurent Kodjikian

**Affiliations:** ^1^Department of Ophthalmology, Croix-Rousse University Hospital, 103 Grande rue de la Croix Rousse, 69317 Lyon Cedex 04, France; ^2^Professor Sahel Department, Rothschild Ophthalmologic Foundation, 25 rue Manin, 75019 Paris, France; ^3^Kleber Retinal Center, 50 Cours Franklin Roosevelt, 69006 Lyon, France; ^4^Department of Ophthalmology, Gui de Chauliac University Hospital, 80 Avenue Augustin Fliche, 34295 Montpellier Cedex 5, France

## Abstract

*Purpose*. To report an association between reticular pseudodrusen, located above the retinal pigment epithelium (RPE), and Early Onset Drusen (EOD) as described using Spectral-Domain Optical Coherence Tomography (SD-OCT). *Methods*. Eight patients (16 eyes) with EOD were examined. EOD were classified into three entities called Large Colloid Drusen (LCD), Malattia Leventinese (ML), and Cuticular Drusen (CD). Best-corrected visual acuity, fundus examination, color fundus photographs, fundus autofluorescence (FAF), fluorescein angiography (FA), indocyanine green angiography (ICGA), and SD-OCT were performed in all study patients. *Results*. Four patients had LCD, 2 had ML, and 2 had CD. Reticular pseudodrusen were observed with SD-OCT in all study patients; all these patients had hyperreflective lesions above and below the RPE. *Conclusion*. Early Onset Drusen appear to be associated with reticular pseudodrusen. SD-OCT is helpful in distinguishing the location of the deposits that are above and below the RPE in EOD. Further studies are needed to understand the role of reticular pseudodrusen in the pathophysiology of EOD.

## 1. Introduction

 “Soft drusen” are defined as deposits located between the retinal pigment epithelium (RPE) and the inner collagenous layer of Bruch's membrane [1]. These lesions usually appear after the age of 50 and are usually associated with AMD [2]. Younger people can have similar deposits called “Early Onset Drusen” (EOD). These lesions have been recently classified into three entities called Large Colloid Drusen (LCD), Malattia Leventinese (ML), and Cuticular Drusen (CD) [3]. Recent papers have described the multimodal morphological features of EOD as deposits classically located under the RPE similar to the soft drusen observed in AMD [4–6]. Reticular pseudodrusen, frequently associated with AMD, have been described using SD-OCT. Our purpose was to report a frequent association of reticular pseudodrusen, located above the RPE, and EOD using Spectral-Domain Optical Coherence Tomography (SD-OCT). 

## 2. Patients and Methods

**Figure 1:**
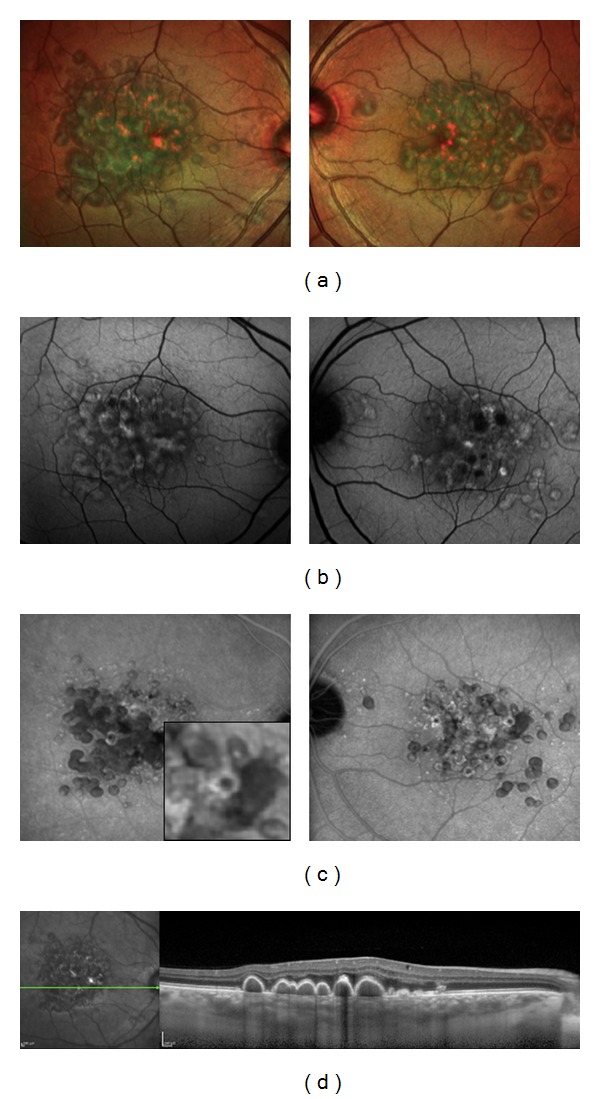
A forty-year-old woman affected by Large ColloId Drusen in both eyes. (a) Multicolor images with large yellowish drusen in the macular area. (b) The larger drusen are hyperautofluorescent in FAF. (c) In the late phase of ICG angiography, LCD are hypofluorescent with a dark centre surrounded by a more fluorescent halo, bordered by a thin hypofluorescent ring (“donut effect”). (d) The colloid drusen are located under the RPE on B-scan OCT.

**Figure 2:**
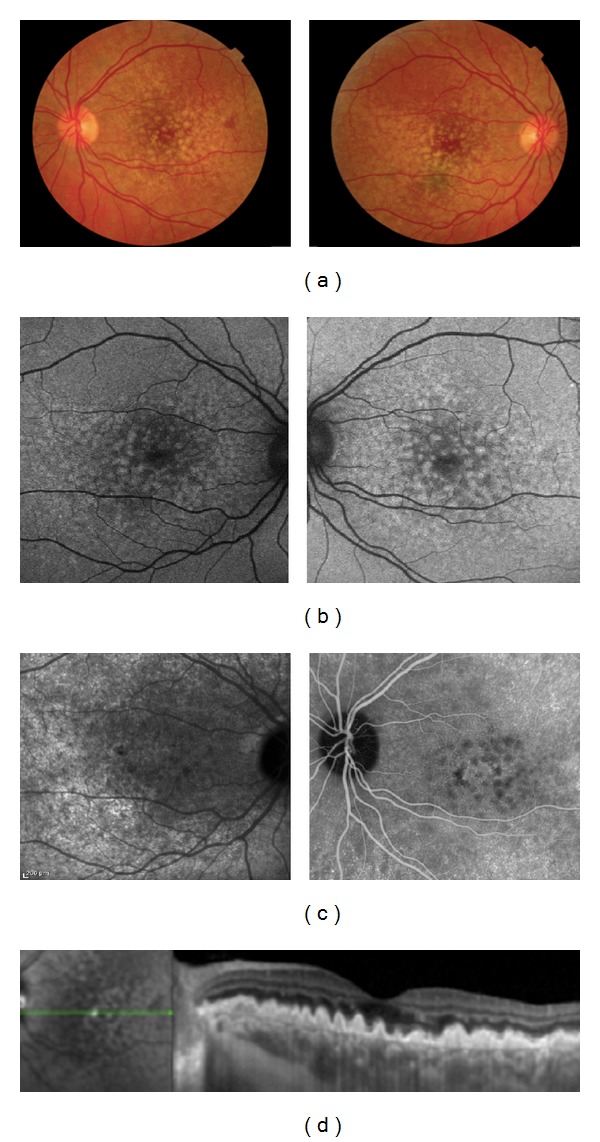
A thirty-five-year-old woman affected by Malattia Leventinese in both eyes. (a) Color fundus photographs with large and small drusen associated with pigmentary changes. The larger drusen are confluent in the macular area while the smaller drusen are located temporally. (b) The larger drusen are hyperautofluorescent. (c) Large drusen are hyperfluorescent with a hypofluorescent halo in the late phase of ICG angiography. (d) The large drusen are located under the RPE on B-scan OCT.

**Figure 3:**
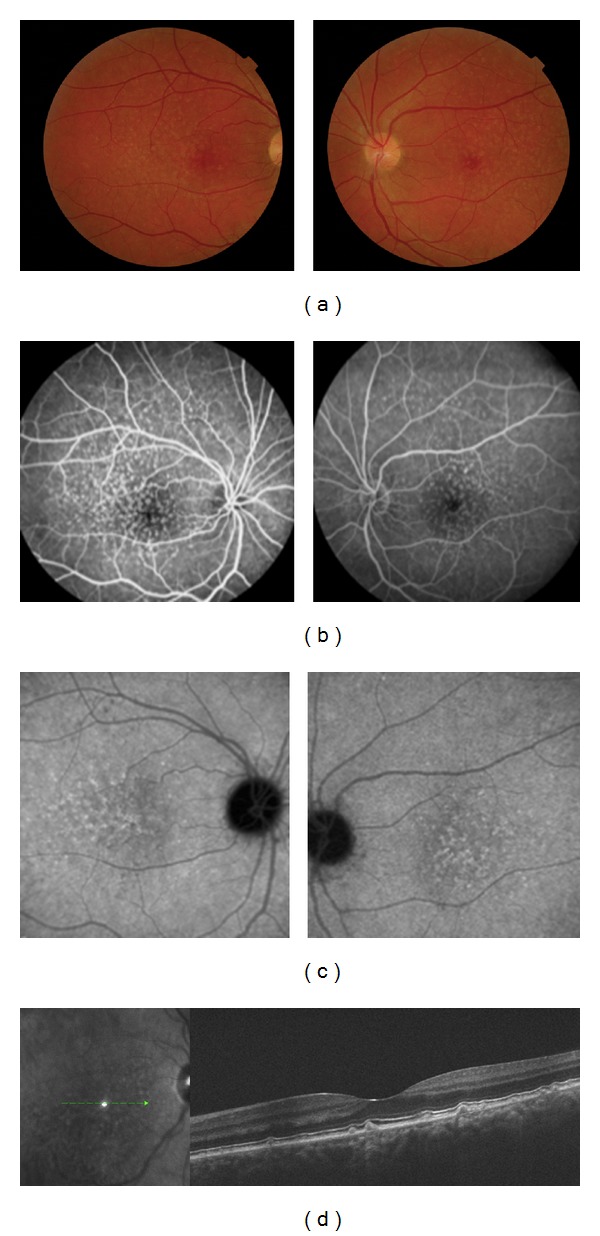
A forty-year-old woman affected by Cuticular Drusen in both eyes. (a) Color fundus photographs with small drusen randomly scattered in the macula and in the middle periphery of the retina. (b), (c) In the late phase of FA and ICG, the drusen are hyperfluorescent with a typical “stars-in-the-sky” appearance. (d) The small drusen are located under the RPE on B-scan OCT.

 Patients with Early Onset Drusen underwent a comprehensive ophthalmologic examination which included best-corrected visual acuity (BCVA), fundus examination, color fundus photographs, multicolor images, fundus autofluorescence (FAF), fluorescein angiography (FA), indocyanine green angiography (ICGA), and Spectral-Domain (Cirrus, Carl Zeiss-Meditec, Dublin, CA, USA; and Spectralis Heidelberg Retinal Angiography OCT, Heidelberg Engineering, Heidelberg, Germany) Optical Coherence Tomography (SD-OCT). Informed consent was obtained as required by the French bioethical legislation (CE_20130319_11_BWF), in agreement with the Declaration of Helsinki for research involving human subjects. The diagnosis of the specific EOD entity was based on fundus biomicroscopy, angiography, and OCT features [3]. Large Colloid Drusen (LCD) are identified on fundus examination as large, bilateral, and yellowish lesions located in the macular area and/or in the periphery of the retina. LCD are hyperautofluorescent and appear in the late phase of ICGA, as a hypofluorescent centre surrounded by a hyperfluorescent halo (Figure 1). This halo is bordered by a hypofluorescent ring, termed as a “donut effect” [5]. In Malattia Leventinese (ML), color fundus photographs show drusen of different sizes: the smaller drusen have a radial distribution whereas the larger drusen are located in the macular area [6]. FAF shows hyperautofluorescence of the larger drusen. In the late phase of ICGA, large drusen are hyperfluorescent, showing a hypofluorescent halo (Figure 2). Cuticular Drusen (CD) are small, round, and yellowish lesions randomly scattered in the macula and in the middle periphery of the retina [4]. In the late phase of FA, the drusen are hyperfluorescent with a typical “stars-in-the-sky” pattern (Figure 3). 

## 3. Results

A total of 8 patients presenting with EOD (4 LCD, 2 ML, and 2 CD) were included in this study (Table 1). All the patients were women, and the mean age at diagnosis was 40 years (35–50).

**Figure 4:**
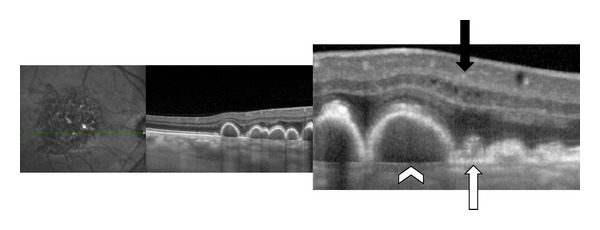
Patient affected by Large Colloid Drusen: SD-OCT shows two different types of deposits. Colloid drusen are convex and observed under the RPE, like soft drusen, with variable reflectivity. Bruch's membrane is visible (white arrowhead). The second type of deposit which is smaller, triangular, and hyperreflective is located above the RPE, like reticular pseudodrusen (white arrow). An abrupt interruption of both the inner band of RPE and IS/OS interface appears at the border of the hyperreflective lesion. Hyporeflective cysts are observed in the inner retina without choroidal neovascularization (black arrow).

**Figure 5:**
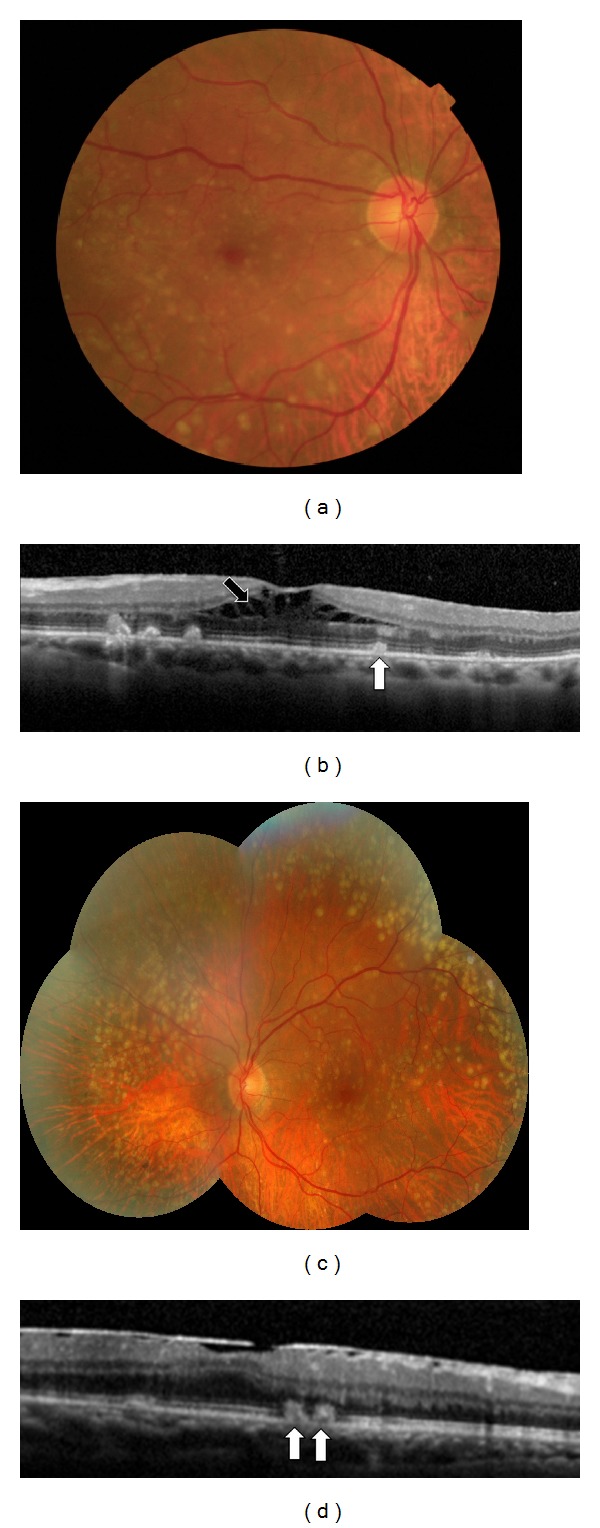
Patient with Large Colloid Drusen in both eyes. (a), (c) Color fundus photographs show large drusen in the macular area and in the periphery of the retina. (b), (d) SD-OCT shows hyperreflective deposits located above the RPE (white arrows). An abrupt interruption of both the inner band of RPE and IS/OS interface appears at the border of these hyperreflective lesions. Hyporeflective cysts are observed in the inner retina along with an epiretinal membrane (black arrow).

**Figure 6:**
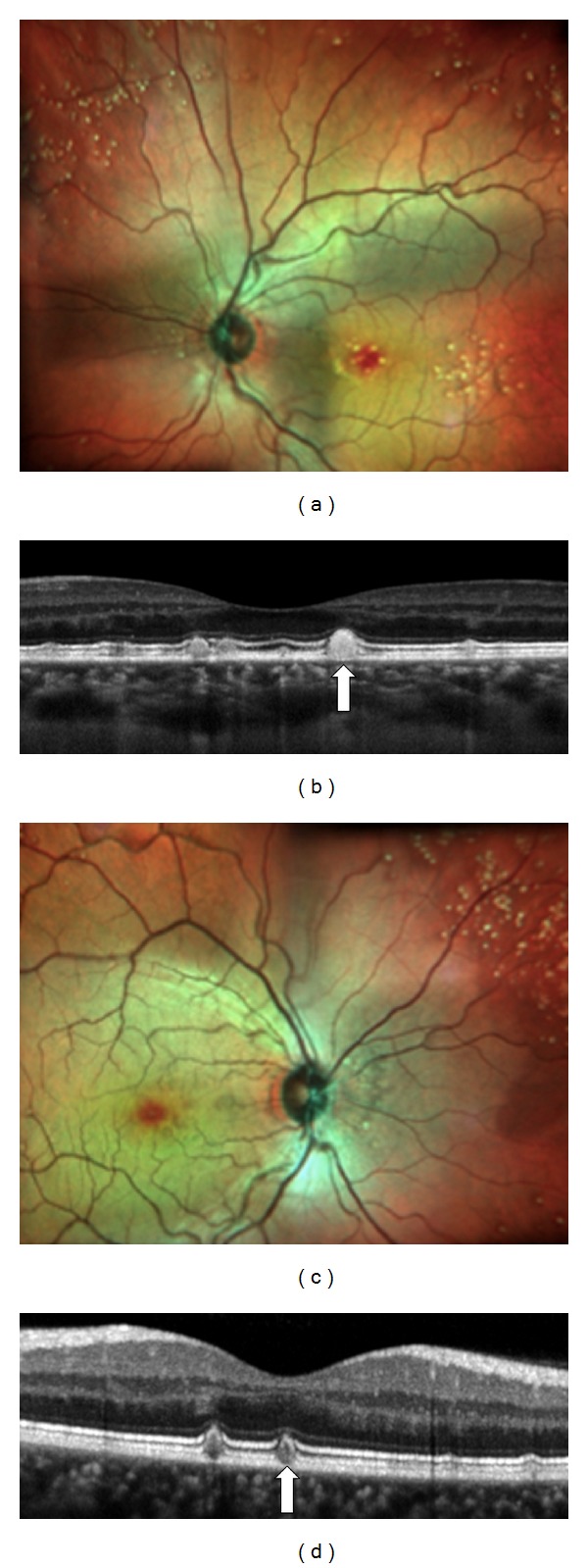
Patient affected by LCD in both eyes. (a), (c) Multicolor images show large drusen located in the macular area and the periphery of the retina. (b), (d) SD-OCT shows hyperreflective deposits with a black center above the RPE and with interruption of both the inner band of the RPE and the IS/OS interface (white arrows).

SD-OCT of the 4 LCD patients showed 2 kinds of deposits at different locations from the RPE: (i) convex colloid drusen with variable reflectivity classically described in LCD were observed between the RPE and Bruch's membrane similar to soft drusen (Figures 4–6), and (ii) round or triangular hyperreflective lesions located between the RPE and the external limiting membrane or the outer plexiform layer (Figures 4–6); the location of these deposits matches the description of reticular pseudodrusen. These subretinal deposits were associated with colloid drusen in all the four patients affected by LCD. Two LCD patients had several hyporeflective cysts in the inner retina without choroidal neovascularization (Figures 4 and 5); one of these patients also had an epimacular membrane (Figure 5).

**Figure 7:**
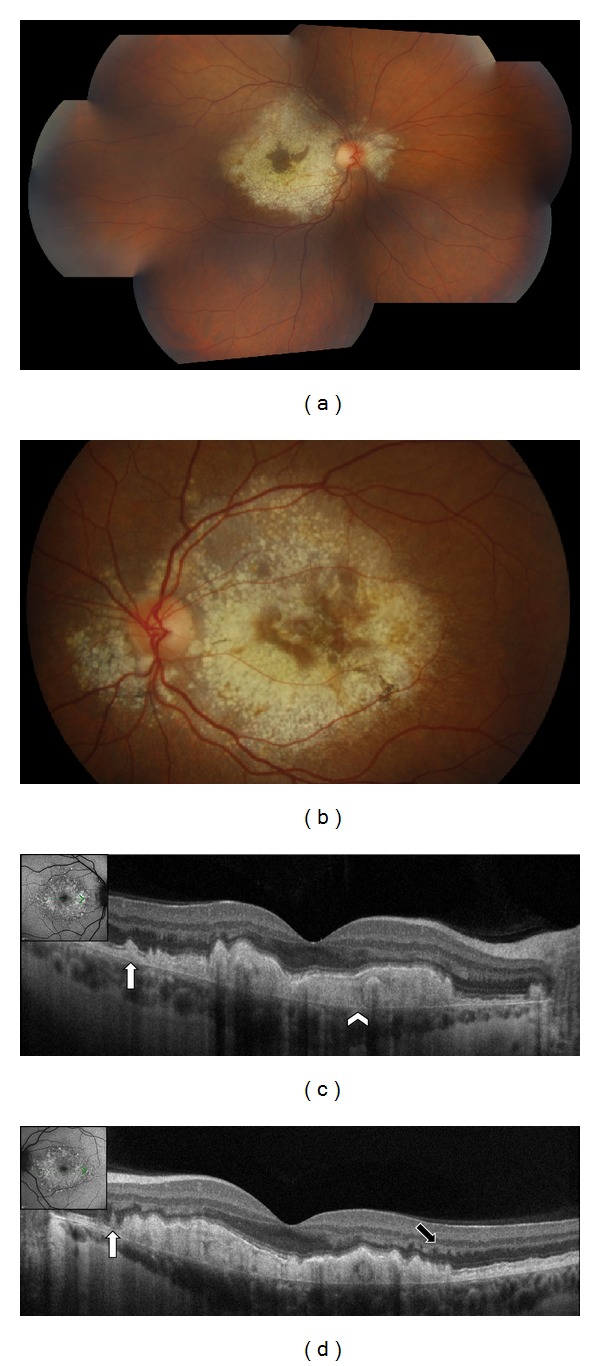
Patient affected by Malattia Leventinese in both eyes. (a), (b) Color fundus photographs show drusen associated with pigmentary changes. (c), (d) SD-OCT shows the larger drusen as diffuse deposition of hyperreflective material under the RPE (white arrowhead). Above the RPE elevation, a hyperreflective haze is observed in the external nuclear layer (black arrow). The smaller drusen are seen above the RPE (white arrows).

**Figure 8:**
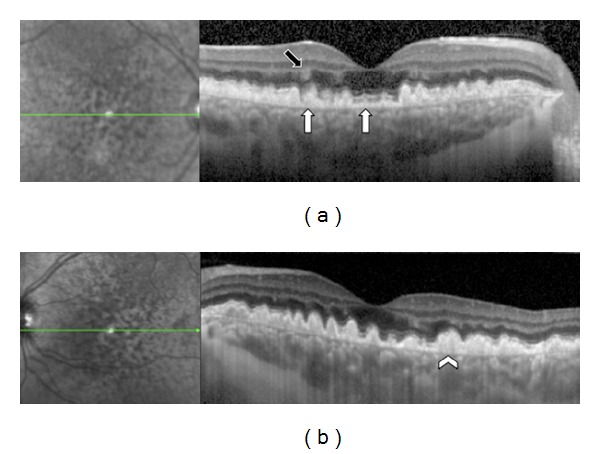
Patient affected by Malattia Leventinese in both eyes. (a), (b) SD-OCT shows the larger drusen as focal deposition of hyperreflective material under the RPE (white arrowhead). Another kind of deposit is seen above the RPE (white arrow). A hyperreflective haze is also seen located in the external nuclear layer close to plexiform layer (black arrow).

Patients with ML also showed 2 distinct kinds of deposits on SD-OCT. The first kind was large and round colloid-like drusen corresponding to focal or diffuse deposition of hyperreflective material between the RPE and Bruch's membrane within the posterior pole. These deposits were associated with a focal dome-shaped or diffuse RPE elevation above Bruch's membrane. The second kind of deposit was a hyperreflective lesion located above the RPE (Figures 7 and 8).

**Figure 9:**
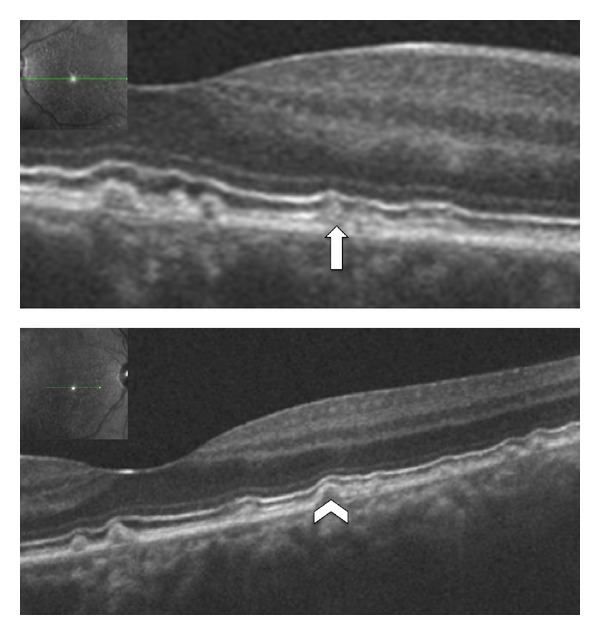
Patient affected by Cuticular Drusen in both eyes. SD-OCT shows some Cuticular Drusen as small RPE elevations (white arrowhead) and others deposits as “sawtooth” RPE elevations above the RPE (white arrow).

In Cuticular Drusen (CD), SD-OCT again showed 2 different kinds of deposits: confluent small “dome-shaped” RPE elevations and “sawtooth” RPE elevations (Figure 9). In the “sawtooth” RPE elevations, the deposits appeared to be above the RPE.

## 4. Discussion

The diagnosis of Early Onset Drusen (EOD) is classically based on fundus examination and angiographic features. Recently, SD-OCT has been used to detect the different EOD entities and to define drusen as deposits observed under the RPE [4–6]. Our study, using SD-OCT, has demonstrated the association between reticular pseudodrusen and EOD. SD-OCT with high resolution allows the histologic imaging of drusen and located them below or above the RPE. In all our 8 study patients, hyperreflective lesions located above the RPE were observed. These images may clinically correspond with reticular pseudodrusen. To our knowledge, this is the first report of this association between reticular pseudodrusen and the three entities of EOD.

Reticular pseudodrusen in AMD have been described by Querques et al. using SD-OCT as hyperreflective material located not below but above the RPE [7]. In 2007, Cohen et al. described these pseudodrusen as a yellowish pattern in the macula frequently associated with AMD [8]. Recently, Curcio et al. reported a pathological description of subretinal drusenoid deposits (SDDs) located above the RPE and associated with photoreceptor perturbation [9]. These subretinal drusenoid deposits have been linked to the phenotype of reticular pseudodrusen. Basal linear deposit (BlinD), on the other hand, corresponds with soft drusen under the RPE. SDD and BlinD were associated and, respectively, detected in 85% and 90% of donor eyes with AMD. More information about the histopathology of SDD would facilitate understanding of its role in AMD pathophysiology and in EOD [10–12] Curcio et al. hypothesized that the RPE is a polarized and bidirectional secretor of lipoproteins participating in lesion formation above and below the RPE [9]. This hypothesis could explain the simultaneous presence of soft drusen and reticular pseudodrusen in patients with EOD and AMD. 

 In conclusion, soft drusen and reticular pseudodrusen seem to be frequently associated in EOD. SD-OCT is helpful in distinguishing the location of the deposits above and below the RPE in EOD. Soft drusen are located below the RPE layers while reticular pseudodrusen are located above the RPE. These two distinct lesions frequently occur simultaneously in young patients affected by hereditary drusen. A new clinical classification of Early Onset Drusen should include the presence of reticular pseudodrusen. Further studies are needed to understand their role in the physiopathology of EOD.

## Figures and Tables

**Table 1:** Data of patients for each type of Early Onset Drusen (EOD) with the number of patients, mean age at diagnosis, sex, and the localization of deposit above or under the retinal pigment epithelium (RPE).

Type of EOD	Number of patients	Mean of age at diagnosis	Gender	Deposit above the RPE	Deposit under the RPE
LCD	4	40	Women	+	+
ML	2	38	Women	+	+
CD	2	42	Women	+	+

LCD: Large Drusen Colloid; ML: Malattia Leventinese; CD: Cuticular Drusen.
